# Evaluation of 2-Thioxoimadazolidin-4-one Derivatives as Potent Anti-Cancer Agents through Apoptosis Induction and Antioxidant Activation: In Vitro and In Vivo Approaches

**DOI:** 10.3390/molecules27010083

**Published:** 2021-12-23

**Authors:** Mohamed S. Nafie, Ahmed I. Khodair, Hebat Allah Y. Hassan, Noha M. Abd El-Fadeal, Hanin A. Bogari, Sameh S. Elhady, Safwat A. Ahmed

**Affiliations:** 1 Department of Chemistry, Faculty of Science, Suez Canal University, Ismailia 41522, Egypt; 2 Department of Chemistry, Faculty of Science, Kafrelsheikh University, Kafr El Sheikh 33516, Egypt; 3 Institute of Biotechnology for Graduate Studies & Research, Suez Canal University, Ismailia 41522, Egypt; omrelansary2010@gmail.com; 4 Department of Medical Biochemistry and Molecular Biology, Faculty of Medicine, Suez Canal University, Ismailia 41522, Egypt; noha_abdelfadeal@med.suez.edu.eg; 5 Department of Pharmacy Practice, Faculty of Pharmacy, King Abdulaziz University, Jeddah 21589, Saudi Arabia; hbogari@kau.edu.sa; 6 Department of Natural Products, Faculty of Pharmacy, King Abdulaziz University, Jeddah 21589, Saudi Arabia; ssahmed@kau.edu.sa; 7 Department of Pharmacognosy, Faculty of Pharmacy, Suez Canal University, Ismailia 41522, Egypt

**Keywords:** apoptosis, molecular docking, PI3K/AKT, 2-thioxoimadazolidin-4-one, in vivo, antioxidant activity

## Abstract

Background: Hepatocellular carcinoma (HCC) is one of the most widespread malignancies and is reported as the fourth most prevalent cause of cancer deaths worldwide. Therefore, we aimed to investigate the probable mechanistic cytotoxic effect of the promising 2-thioxoimidazolidin-4-one derivative on liver cancer cells using in vitro and in vivo approaches. The compounds were tested for the in vitro cytotoxic activity using MTT assay, and the promising compound was tested in colony forming unit assay, flow cytometric analysis, RT-PCR, Western blotting, in vivo using SEC-carcinoma and in silico to highlight the virtual mechanism of action. Both compounds **4** and **2** performed cytotoxic effects against HepG2 cells with IC_50_ values of 0.017 and 0.18 μM, respectively, compared to Staurosporine and 5-Fu as reference drugs with IC_50_ values of 5.07 and 5.18 µM, respectively. Compound **4** treatment revealed apoptosis induction by 19.35-fold (11.42% compared to 0.59% in control), arresting the cell cycle at G2/M phase. Moreover, studying gene expression that plays critical roles in cell cycle and apoptosis by RT-PCR demonstrated that compound **4** enhances the expression of the pro-apoptotic genes p53, PUMA, and Caspase 3, 8, and 9, and impedes the anti-apoptotic Bcl-2 gene in the HepG2 cells. It can also inhibit the PI3K/AKT pathway at both gene and protein levels, which was reinforced by the in silico predictions of the molecular docking simulations towards the PI3K/AKT proteins. Finally, in vivo study verified that compound **4** has a promising anti-cancer activity through activating antioxidant levels (CAT, SOD and GSH) and ameliorating hematological, biochemical, and histopathological findings.

## 1. Introduction

Cancer is a fundamental public health concern [1]. The six most common cancers found worldwide are liver, colon, breast, prostate, stomach, and lung. Hepatocellular carcinoma (HCC) is a complication commonly associated with chronic liver disease, and globally HCC is categorized as the fourth cause of death and the sixth in incidence [2]. In addition, the highest rate of HCC incidence is found in Africa and Asia regions with a high frequency of HBV and HCV infection [2,3]. Therefore, it is urgent to find adequate care or treatment for patients with end-stage liver disease and develop anti-HCC drugs. Despite ongoing advancement in understanding the biological basis of hepatocellular carcinoma (HCC) spread, clinical disease management remains a significant challenge. Hepatic cell carcinogenesis is related to the PI3K/Akt/mTOR pathway’s dysregulation, a prototypical survival pathway [4]. Phosphatidylinositol 3-kinase (PI3K)/Akt is considered a critical cellular signaling pathway that plays a vital role in regulating cell proliferation, growth, cell size, metabolism, and motility [5]. In addition, it improves understanding of the mechanisms involved in the evolution of cancer and provides a new target molecular therapy for HCC [6]. Many studies found that the PI3K/Akt pathway is enhanced in cancer, so inhibiting this pathway may contribute to cancer cell regression and clinical and preclinical trials.

**Figure 1:**
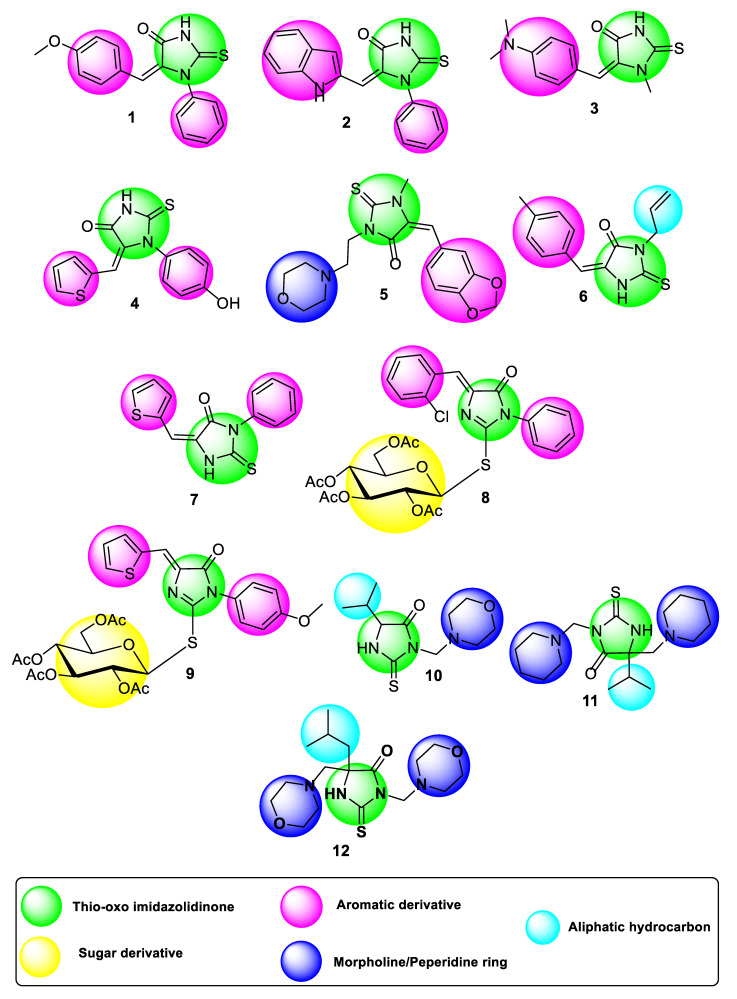
Structures of the investigated 2-thioxoimadazolidin-4-one tested compounds (**1**–**12**) with highlighted substitutions.

2-thioxoimadazolidin-4-one derivatives structure, through their five heterocyclic rings, were the most popular scaffolds to produce different agents with various pharmacological activities [7]. Discovery of new and efficient anti-cancer agents in medicinal chemistry remains a significant and challenging task. Therefore, establishing new anti-cancer drugs with more successful therapeutic approaches and well-pharmacokinetic properties is of vital importance [8]. The purpose of this paper is to assess the cytotoxic activity of 2-thioxoimadazolidin-4-one derivatives (Figure 1) in a human liver hepatocellular carcinoma cell line (HepG2 cell line), to study the apoptotic effect of the most promising 2-thioxoimadazolidin-4-one derivative and to investigate its effect on the PI3K/AKT pathway.

## 2. Results

### 2.1. Cytotoxic Activity

As demonstrated in Table 1, almost all compounds displayed relevant cytotoxic activities with IC_50_ ranging from 0.017 to 8.9 µM. Two compounds, **4** and **2**, showed the potent cytotoxic activity against HepG2 cells with IC_50_ values of 0.017 and 0.18 µM, respectively, which were more potent than Staurosporine and 5-FU as reference drugs with IC_50_ values of 5.07 and 5.18 µM, respectively. In comparison to compound **4** as the most promising activity, compounds **1** and **10** exhibited the lowest activity with the highest IC_50_ values of 27.82 and 64.78 µM, respectively. Based on the safety of compound **4** toward normal liver cells (THLE-2) with the high IC_50_ value, this indicates its high selectivity toward cancer cells. However, the MTT technique was used to indicate the cytotoxicity of tested derivatives, which encouraged us to study further the cellular and molecular mechanisms underlying such potent cytotoxicity of one of the promising compounds.

### 2.2. Colony Forming Assay

**Figure 2:**
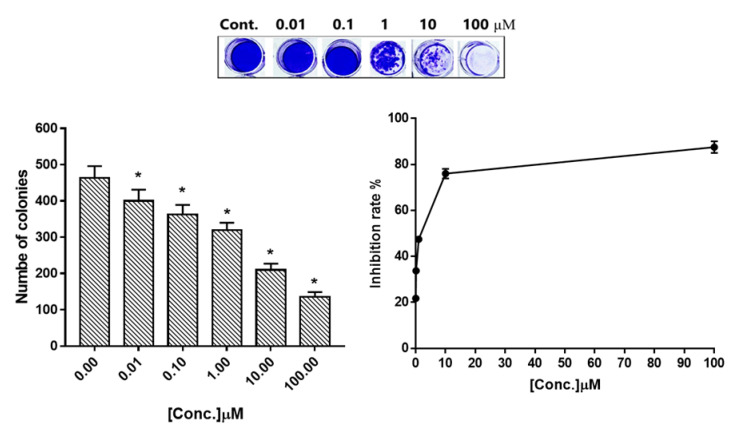
Representative images of colony numbers in control and treated HepG2 cells with different concentrations (0.01, 0.1, 1, 10, 100 μM) of compound **4**. Values are represented by mean ± SE (*n* = 3 independent trials). * Significant difference between control and treated HepG2 cells with different concentrations using ANOVA (*p* ≤ 0.05) using SPSS Version 22.

The CFU assay has been used extensively to study the effects of compound **4** on HepG2 cells. Therefore, HepG2 cells were treated with compound **4** (IC_50_ = 0.017 μM) for 48 h to test the compound ability to proliferate and form small colonies. As shown in (Figure 2), fewer and smaller colony cells were observed after the cells were treated with compound **4**, and the colony inhibition rate reached around a high of 88% at 100 μM, with 136 colonies compared to 464 in control. 

### 2.3. Apoptotic Investigation

#### 2.3.1. Compound **4** Induces Cell Cycle Arrest in G2/M Phase in HepG2 Cells

**Figure 3:**
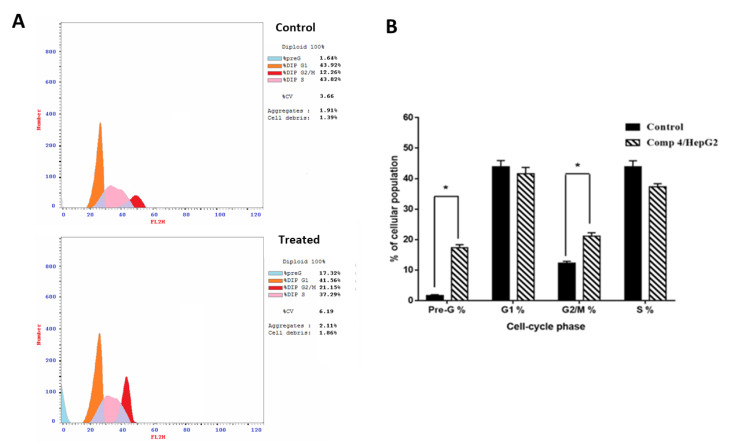
(**A**) Cytogram of cell cycle analysis of compound **4** treatment (IC_50_ = 0.017 μM) in HepG2 cells compared to untreated control and (**B**) bar representation for the untreated and treated HepG2 cells in each phase. Values are represented by mean ± SE (*n* = 3 independent samples per group). * Significant difference between control and treated groups using unpaired Student’s *t*-test (*p* ≤ 0.05) using SPSS Version 22.

Studying the cell cycle stages by measuring DNA content is an important test to help evaluate the effect of compound **4** on cell division and may reflect the mechanism through which compound **4** affects those cells. Induction of cell cycle arrest is another commonly proposed mechanism for anti-cancer drug cytotoxicity and apoptosis. We found that compound **4** inhibited HepG2 cancer cell cycle progression by inducing G2/M phase cell cycle arrest by 21.15% compared to 12.26% in control. Moreover, this is accompanied by a significant increase at Pre-G1 phase. Therefore, compound **4** induced an arrest in the Pre-G1 and G2/M phases of the cell cycle (Figure 3). Cytotoxicity by tested molecules that induce cell arrest usually results in cell death in cancer cells.

#### 2.3.2. Compound **4** Treatment Induced Apoptosis in HepG2 Cells

**Figure 4:**
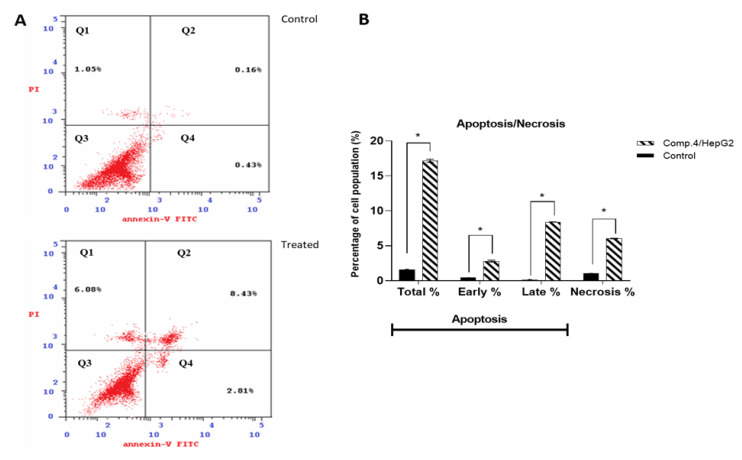
(**A**) Compound **4** induces apoptosis using FITC/annexin-V-FITC/PI differential apoptosis/necrosis. Quadrant charts show Q1 (necrotic cells, AV−/PI+), Q2 (late apoptotic cells, AV +/PI+), Q3 (normal cells, AV−/PI−), Q4 (early apoptotic cells, AV+/PI−). (**B**) Bar representation of apoptosis percentages of untreated and treated HepG2 cells with compound **4** (IC_50_ = 0.017 μM) in early, late, and total. Values are represented by mean ± SE (*n* = 3 independent samples per group). * Significant difference between control and treated groups using unpaired Student’s *t*-test (*p* ≤ 0.05) using SPSS Version 22.

We investigated the ability of the most active compound (compound **4**) to induce the apoptotic program. DNA content data implied arrest of the preG1 and G2/M phase cell cycle under treatment with compound **4**, but in such experiments, it is impossible to determine if this cell death is apoptotic or necrotic. An essential technique for distinguishing apoptotic cell death from necrotic cell death is annexin V/PI staining. The percentage of apoptosis was determined by using flow cytometry. Human hepatoma HepG2 cells were treated with Compound **4** (IC_50_ = 0.017 μM), and the annexin V-FITC/PI staining test was used to quantify apoptotic cells. The results are shown in Figure 4. It was found that treatment with compound **4** increased the proportion of apoptotic (annexin positive) cells in HepG2 cells at 11.42% compared to 0.59% in control. Compound **4** caused cell necrosis and deaths with 6.08% compared to 1.05% in the control cells.

#### 2.3.3. Compound **4** Treatment Induced Up and Downregulation of Apoptosis-Related Genes in HepG2 Cells

The balance between cell death and division is important for maintaining cell death. The mechanisms of extrinsic and intrinsic apoptotic signaling caused the creation of novel target treatments, which can stimulate cell death to recognized cytotoxic agents. Intrinsic pathways include activating P53, pro-apoptotic genes PUMA, Bax, and cascades of Caspase 3 and 9, with inhibition of anti-apoptotic Bcl-2 family and PI3K/AKT genes, while extrinsic pathways contain death receptors and activate cascades of Caspase 8. Gene expression analysis was performed for both intrinsic and extrinsic genes controlling apoptosis.

**Figure 5:**
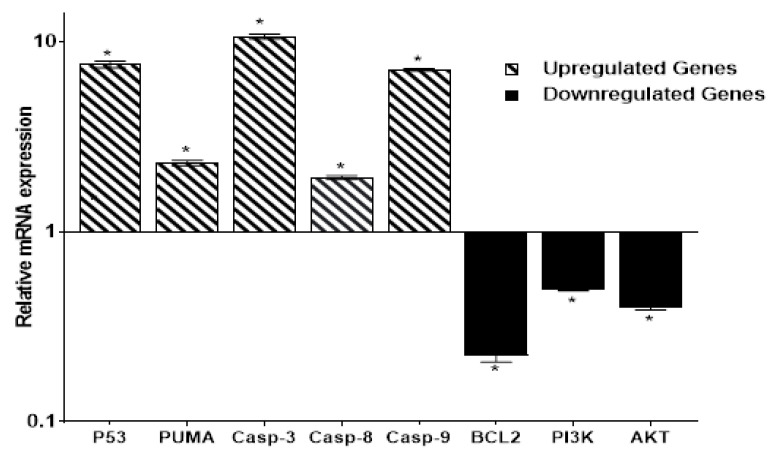
Gene expression of apoptotic-related genes in control and treated HepG2 cells with compound **4** (IC_50_ = 0.017 μM) using RT-PCR. Untreated control is represented by a black line (Fold change = 1). Values are represented by mean ± SE (*n* = 3 independent sample per group). * Significant difference between control and treated groups using an unpaired Student’s *t*-test (*p* ≤ 0.05) using SPSS Version 22.

Compared to control cells, we found that the treated HepG2 cells showed upregulation for P53, PUMA, and Caspase 3, 8, and 9 by 7.61-fold, 2.31-fold, 10.71-fold, 1.98-fold, and 7.14-fold, respectively. A molecular marker of programmed cell death is the activation of caspases; Caspase 3 is a common caspase responsible for most of the cell death process and is activated by upstream initiator caspases such as Caspase 8 and 9. In contrast, the anti-apoptotic BCL-2 gene expression was significantly reduced by 0.25-fold. Furthermore, the expression of both PI3K and AKT genes was reduced by 0.497-fold and 0.38-fold, respectively, suggesting that both intrinsic and extrinsic apoptosis were present in the treated HepG2 cell line. Inhibition of the PI3K/AKT pathway has been identified as a promising cancer therapeutic target (Figure 5).

#### 2.3.4. Compound **4** Induced Up and Downregulation of Target Proteins in HepG2 Cells

**Figure 6:**
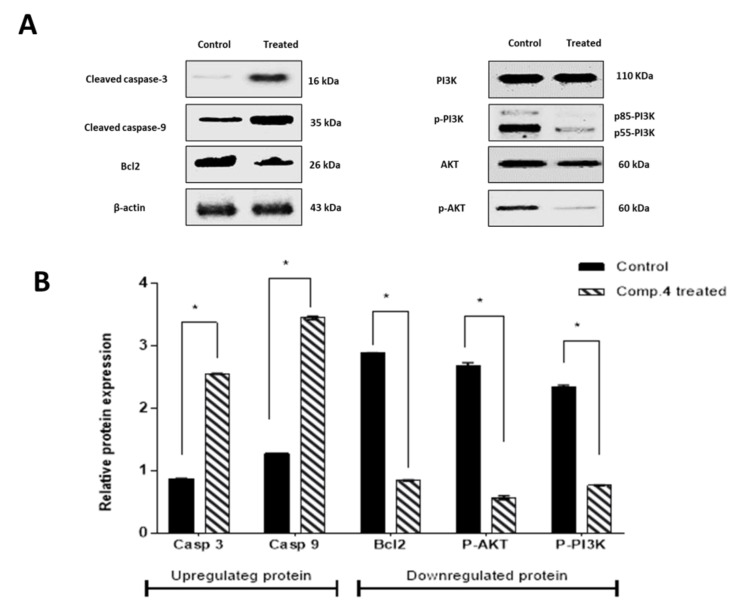
Effects of compound **4** on the expression of apoptosis-related proteins. Band widths (**A**) and quantification (**B**). The protein expression of Caspase 3 and 9, Bcl-2, PI3K, and AKT in HepG2-treated cells and control. Data were normalized using β-actin. Values are represented by mean ± SE (*n* = 3 independent sample per group). * Significant difference between control and treated groups using an unpaired Student’s *t*-test (*p* ≤ 0.05) using SPSS Version 22.

By Western blot technique, we measured the expression level of Caspase 3 and 9, Bcl-2, PI3K, and AKT proteins establishing the apoptosis induction mechanism caused by compound **4** in HepG2 cells. PI3K is a potential and drug-enabled target for cancer treatment. PI3K/Akt is recognized as one of the most important signaling pathways in regulating cell growth, proliferation, and apoptosis, and AKT is a major downstream target of PI3K. We investigated whether compound **4** activates the PI3K/AKT pathway and whether this pathway plays a central role in apoptosis via compound **4**. Western blot analysis showed that PI3K, AKT, and BCL2 levels were decreased in compound-**4**-treated HepG2 cells compared with untreated control. In contrast, Caspase 3 and 9 proteins were upregulated, which is indicated by visualizing both thick and thin bands (Figure 6A). Their relative quantification data are shown in (Figure 6B). These results agreed with RT-PCR results of pro-apoptotic gene upregulation and anti-apoptotic gene downregulation. 

### 2.4. In Vivo Study

#### 2.4.1. Anti-Tumor Potentiality

**Figure 7:**
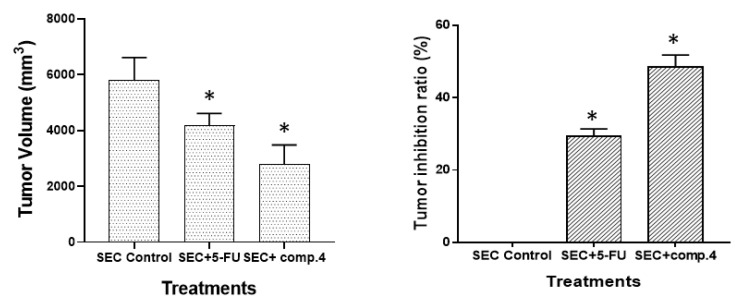
Tumor volume (mm3) and inhibition ratio (%) in SEC-bearing mice treated with a dose of 6 mg/kg of compound **4** and 5-FU. * Significant difference between control and treated groups using ANOVA (*p* ≤ 0.05) using SPSS Version 22.

Figure 7 shows the tumor volume and inhibition ratio after 20 days of treatment. Tumor volume was significantly increased by inoculation of solid Ehrlich carcinoma (SEC cells) into mice, and after treatment with compound **4**, all treated SEC mice had a significant decrease in tumor volume The tumor volumes on the final day of the experiment were measured as (5802 mm^3^) in the SEC-treated group, but in compound-4-treated mice, the tumor volume was significantly reduced (2766 mm^3^), as well as the 5-FU group (4177 mm^3^). In addition, compound **4** caused a promising increase in the tumor inhibition ratio by 48.4%, compared to 29.28% in the 5-FU-treated mice (Figure 7). 

#### 2.4.2. Hematological Parameters

Table 2 showed the effect of compound **4** treatment on hematological parameters of SEC tumor-bearing mice. Treatment of SEC mice with compound **4** exhibited a non-significant increase in hemoglobin level and RBCs count and a significant decrease in WBCs count compared to other groups, and the values of these parameters in SEC treated with compound **4** were close to their levels in the normal control group. All these parameters indicate the safety of compound **4** on hematological parameters by retaining values near normal.

#### 2.4.3. Biochemical Parameters

As shown in Table 3, compound **4** treatment showed non-significant decrease in serum ALT and AST levels, while it increased total protein, urea and creatinine levels in SEC mice compared to other groups. These findings indicate the safety of compound **4** on the liver and kidneys.

#### 2.4.4. Compound **4** Treatment Induced Antioxidant Activation

The levels of oxidants and antioxidants in both untreated and 4-treated SEC-bearing mice are summarized in Table 4 in comparison to a normal control group. Results showed that oxidative stress was increased, while SEC mice had significantly reduced antioxidants because of cancer oxidative stress. With treatment of SEC-bearing mice, compound **4** significantly reduced MDA and NO levels to 73.36 (nmol/g.tissue) and 42.38 (U/g tissue), respectively, compared to 98.39 and 79.69, respectively, in the SEC control group. On the other hand, antioxidant levels were significantly increased; it was observed that GSH was increased to 39.27 (mg/g.tissue) compared to 18.23 (mg/g.tissue), CAT was increased to 46.9 (U/g.tissue) compared to 21.69 (U/g.tissue), and SOD was increased to 48.69 (U/g.tissue) compared to 27.36 (U/g.tissue).

#### 2.4.5. Histopathological Examinations

##### Effect of Compound **4** on the Liver Tissue of SEC-Bearing Mice

**Figure 8:**
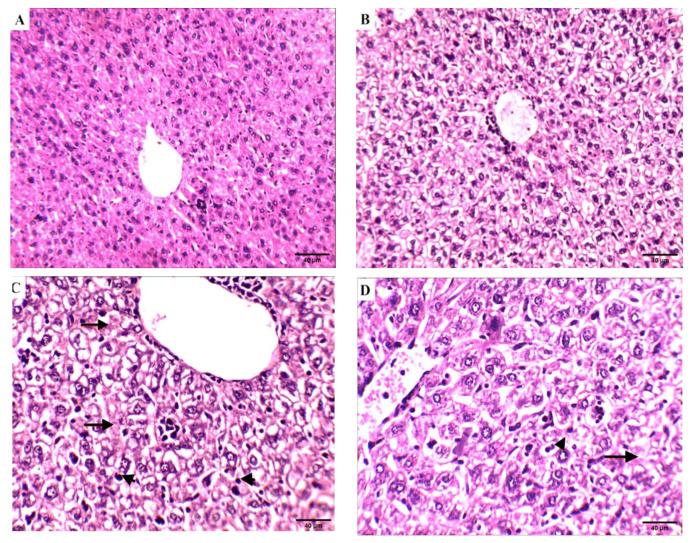

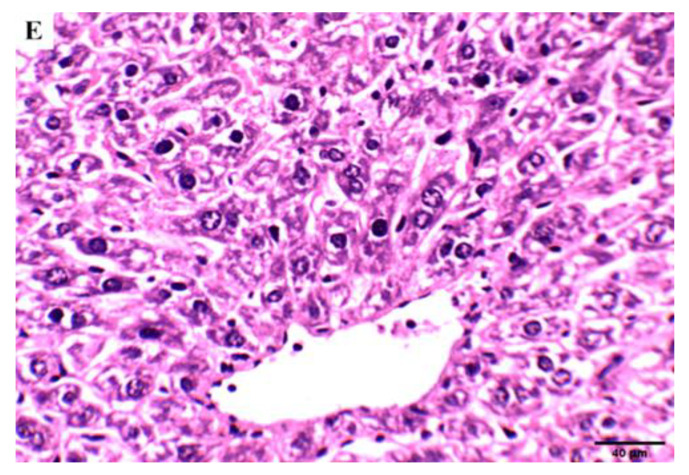
Effect of compound **4** on the liver tissue of SEC-bearing mice: (**A**) normal control, (**B**) normal group treated with compound **4.** (**C**) SEC control group, shows nuclear pyknosis (arrowheads) and karyolysis (arrows). (**D**) SEC mice treated with 5-FU still exhibit hydropic degeneration of hepatocytes, nuclear pyknosis (arrowheads), and karyolysis (arrows). (**E**) SEC mice treated with compound **4** (H&E stains, magnification ×200). Quantification data of the H&E analysis of liver are supported in Supplementary Table S2.

The effect of compound **4** treatment on hematological and biochemical parameters was demonstrated through liver histopathological examination (Figure 8). Liver tissue from normal control mice showed a normal anatomical structure of liver lobes and polyhedral hepatocytes with a nucleus and granular cytoplasm. Hepatocytes arranged in alternating strands with blood sinusoid forming a network around the central vein, and liver sections of normal control mice treated with compound **4** showed normal-looking liver lobules; some hepatocytes showed a hydropic degeneration and a balloon degeneration. On the other hand, degenerated balloons were visible in the SEC control group with cytoplasmic vacuolation, intranuclear cytoplasmic inclusions, and absence of cell boundaries, and liver tissue loss of membrane integrity leads to cell fusion. Hydropic degeneration of the hepatocytes and other hepatic cells had nuclear pyknosis (arrowheads) and karyolysis. Hydropic degeneration of hepatocytes, nuclear pyknosis, and karyolysis still occurred in the 5-FU group in the SEC group. On the contrary, the SEC group + compound **4** group showed liver lobules looking normal. In addition, few hepatocytes showed hydropic degeneration and activated Kupffer cells. These findings support the results previously seen after tumor volume measurement, which indicates that mice treated with compound **4** were more efficient at inhibiting tumor growth.

##### Effect of Compound **4** on the Kidney Tissue of SEC-Bearing Mice

**Figure 9:**
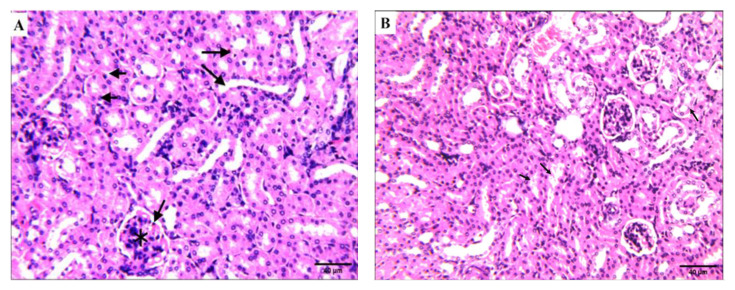

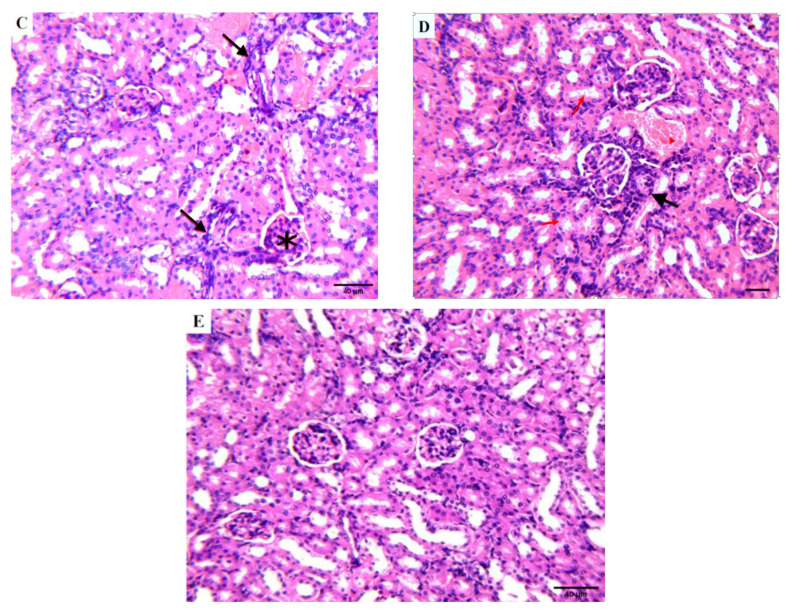
Effect of compound **4** on the kidney tissue of SEC-bearing mice. (**A**) Normal control group shows intact renal corpuscle of the glomerulus (asterisk) and bowman’s capsule with urinary space (arrowhead) surrounded with proximal (short arrows) and distal convoluted tubules (long arrows). (**B**) Normal group treated with compound **4** shows very few tubules and evidence of acute tubular injury (black arrows). (**C**) SEC control group mice show inflammatory infiltration in interstitial spaces (arrows), renal corpuscles show congestion and hypercellularity (asterisk), glomerular and intratubular hyperemia. (**D**) SEC mice treated with 5-FU: few tubules show evidence of renal acute tubular injury ATI (red arrows), focal area of hemorrhage (red arrowhead), and interstitial inflammation (white arrow). (**E**) SEC mice treated with compound **4** (H&E stains, magnification × 400). Quantification data of the H&E analysis of kidney are supported in Supplementary Table S2).

Histopathological changes in the kidney structure in the study groups are shown in (Figure 9). The structure of glomeruli and renal tubules in the control group was normal, and in the normal group treated with compound **4** (Figure 9A,B). These results suggest that compound **4** is safe in normal cells. Kidney sections in inoculated mice with solid Ehrlich carcinoma showed various pathological changes in glomeruli and renal tubules, such as significant damage and degeneration of kidney tissue, glomerular atrophy, the Malpighian corpuscles that lost their characteristic configuration, and inflammatory infiltration in interstitial spaces. On the other hand, compared with the SEC group, the kidney sections of SEC mice treated with 5-FU showed a moderate improvement and treatment of the histological structure of the kidney (Figure 9D), while the SEC mice treated with compound **4** showed almost normal, intact renal corpuscles and tubules (Figure 9E).

### 2.5. In Silico Studies

#### 2.5.1. Molecular Docking

**Figure 10:**
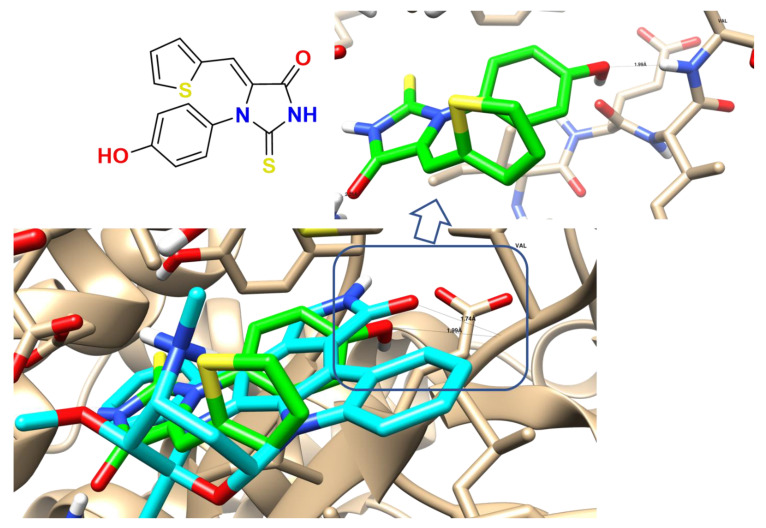
Binding disposition and interactive pose compound **4** (green) and the co-crystalized ligand (cyan) of the PI3K protein.

In this study, to elucidate the virtual mechanism of compound **4** binding, we used a structural drug design tool for docking it inside PI3K and AKT active sites (PDB: 1E8Z and 3QKK) to prove the signaling pathway of the PI3K/AKT. Table 5 summarizes the interaction between compound **4** inside both proteins and illustrates its ligand–receptor interactions, with its binding energies and 2D interactions. Moreover, compound **4** shows that the inside AKT protein formed lipophilic interactions with lipophilic amino acids: Ile 879, Ile 963, Ile 881, Val 882, Ile 831, Met 804, Phe 961, Ala 885, Trp 812, and Met 953, as shown in Figure 10. 

Regarding 1E8Z, the co-crystallized ligand (Staurosporine), and one hydrogen bond with the Val 228 as the interactive key amino acid, compound **4** was docked with binding energy −18.58 Kcal/mol and formed one HB as the HB-donor through its hydroxylic group with Val 228. Additionally, it formed, through its aromatic moieties, arene–arene interaction with Tyr 867 and arene-cation with Lys 890. Regarding 3QKK, compound **4** was docked inside its active site with binding energies −10.97 Kcal/mol, and formed one HB as the HB-donor through its NH group with Asp 292. Therefore, the docking studies of compound **4** inside the two tested proteins indicated that compound **4** showed promising binding activity as PI3K/AKT inhibitors. This agrees with both gene and protein inhibition levels, which may be the proposed anti-liver cancer mode of action. 

#### 2.5.2. ADME Pharmacokinetics

Bioinformatics studies were carried out to determine physicochemical properties and drug-like properties tested for the future drug candidates with Lipinski’s Ro5 [9]. For good intestinal pharmaceutical absorption, the blood–brain barrier (BBB) should be as low as 90 Å2, and the topological polar area of the surface (TPSA) values should be as low as 140 [10]. All of the compounds investigated were highly permeable and absorbed. As shown in Table 6, compounds had 1,2 donors and 4–6 acceptors for hydrogen bonding. In addition, all of the compounds tested have log P values ranging from 0.69 to 2.88, so cell membranes tolerate them well. To control for conformational changes and oral bioavailability, the ratable bond number (nrotb) should be ≤10. All compounds tested have 2–6 nrotb. In addition, compound **4** showed good absorption from the gastrointestinal tract according to the BOILED Egg model (Supplementary Figure S2).

## 3. Discussion 

Cancer significantly affects mortality and morbidity, and many chemotherapies have a low response rate, pushing scientists to identify novel compounds that can suppress cancer progression. One of the main organic scaffolds useful in the various physiological and pharmaceutical eras is imidazolidine. 2-thioxo-imidazolidine-4-one is a sulfur analogue of hydantoin in which one carbonyl group is replaced by a thiocarbonyl group. 2-thioxo–imidazoline-4-one belongs to a heterocyclic compound with a wide range of biological and pharmacological properties. Of the known thiohydantoins, 2-thioxo-imidazolidine-4-one is particularly well known for its wide applications as being hypolipidemic [11], anti-tumor [12], antimutagenic [13], antituberculosis [14], antithyroid [15], antiviral [16], and antimicrobial [17,18]. Changes to the steroid backbone, which change physiology and lead to the development of new, biologically active molecules, are a primary objective of today’s steroid chemistry [19]. New 2-thioxo-imidazolidin-4-one derivatives contain pyrimidine, diaryl, triaryl, cycloalkyl amines, and glucopyranosyl substituents and their aryl and halogen and alkyl derivatives, and other substituents with varied monocyclic (thiadiazole, sulfadiazole) heterocycles linked directly or through alkyl chain with the imidazolidine nucleus [20]. Several published articles and patents discussed the discovery and development of various promising 2-thioxo-imidazolidin-4-one derivatives in cancer therapy [21].

Apoptosis is a key regulator of normal tissue homeostasis, and apoptosis dysregulation is a key cancer inhibition mechanism [22,23]. We revealed a cytotoxic and apoptotic effect of compound **4** on a HepG2 cell line. Our results are consistent with a previous study [24], in which cytotoxicity of some 2-thioxoimidazolidin-4-one derivatives were studied against HepG2 cancer cell line with promising IC_50_ values. As a result, apoptosis stimulation is regarded as a critical cellular event that contributes to the therapeutic effectiveness of anti-cancer drugs. Indeed, many anti-cancer drugs work by inducing apoptosis in cancer cells and preventing the spread of disease [25]. In this study, flow cytometry, gene and protein expression analysis were used to determine apoptosis.

Compared with the control group, the significantly increased number of early apoptotic cells (annexin V positive/PI negative) in the compound **4** treatment group further indicated that compound **4** induced apoptosis and blocked the HepG2 cancer cell cycle progression. Additional mechanistic studies showed that these derivatives induced cell cycle arrest in the G2/M phase [26]. Our results of induction of apoptosis in the treated HepG2 cell line were consistent with previous studies [27], which demonstrated the apoptosis activity of several 2-thioxo-imidazolidine-4-one derivatives in the HepG2 cell line. Oxidative stress is an essential part of cancer development, progression, and inhibition of tumor growth [28]. GSH and CAT deficiency are linked to mitochondrial malfunction and cellular death [29]. MDA, a lipid peroxidation intermediate, directly represents cellular oxidative damage [30]. In this work, compound **4** treatment caused an increase in intracellular MDA levels as well as a decrease in GSH levels in HEPG2 cells. Apoptosis usually occurs by the mitochondrial (intrinsic) pathway and/or the death receptor (extrinsic) pathway [31]. The Bcl-2 family of proteins are critical regulators of the intrinsic pathway. The balance between anti-apoptotic (Bcl-2) and pro-apoptotic groups influences the intrinsic apoptotic pathway. Enhancement of pro-apoptotic subgroups over anti-apoptotic proteins can induce mitochondria to lose ΔΨm and release cytochrome c, thereby activating the intrinsic apoptotic pathway. The expression of Bcl-2 family proteins was measured to investigate whether the mitochondrial apoptotic actions contributed to the apoptosis induced by compound **4**. The result revealed that apoptosis induced by compound **4** was controlled by a mitochondrial pathway, which was verified by downregulation of Bcl-2 proteins, supporting the results of flow cytometric analysis of apoptosis. Therefore, compound **4** induced apoptosis in HEPG2 cells possibly by activation of the mitochondria-dependent pathway including Bcl-2 family proteins. Caspases are the vital machineries in the implementation of apoptosis, and serve essential roles in the initiation and completion of mitochondria-mediated apoptosis [32]. To understand the action mechanism of compound **4** activated apoptosis in HEPG2 cells, the role of the mitochondrial-mediated apoptotic pathway involving cleaved Caspase 3 and cleaved Caspase 8 was investigated by Western blot analysis.

Apoptosis was shown to be driven by 2-thioxo-imidazolidin-4-one derivatives that were ensured by gene and protein expression analysis which revealed a reduced Bcl-2 anti-apoptotic protein expression, PI3K and AKT, and actively increased the levels of Caspase 9 and Caspase 3 [33]. The PI3K/AKT signaling path is critical in regulating several cell processes in physiological and pathophysiological conditions such as cell death and proliferation. Abnormal activation of this pathway is implicated in cell transformation, tumorigenesis, cancer development, and drug resistance in cancer cells [34].

The results agree with the suggested apoptotic pathway for anti-cancer activity through PI3K/AKT inhibition [35,36]. Thus, inhibiting or blocking the PI3K/AKT pathway has been identified as a promising therapeutic target in cancer therapy [37]. In addition, in an in silico study, the compound could bind properly with PI3K and AKT, validated by downregulation of Bcl-2, PI3K, and AKT. As a result, inhibiting this pathway is an important strategy in cancer chemotherapy [38]. Due to the important role of the PI3K pathway in the growth of normal cells and the response of cells to stress, the main challenge in developing PI3K pathway targeting agents is to identify inhibitors with available therapeutic indexes [39].

In addition, the data obtained in this study indicate that the administration of compound **4** in SEC-bearing mice slightly altered the hematologic picture compared with the 5-FU group. Previous studies depicted that the hematological changes accompanied the progression of cancer compared to normal mice; for example, there is a gradual reduction in hemoglobin level, RBCs count, and gradual increase in WBCs count. In addition, myeloid destruction and anemia have been commonly observed in cancer. The major challenges of cancer chemotherapy with conventional drugs are myeloid destruction and anemia [40]. Our data showed that compound **4** reversed the SEC-induced alteration in hematological parameters by elevation of hemoglobin level and total RBCs count and decrease of elevated total WBCs count. These results demonstrate that compound **4** lacks one of the most common side effects of cancer chemotherapy.

One best dose was selected to determine its efficacy against the induced solid tumor model, and this dose was effective in decreasing the solid tumor volume compared to control. Another important undesirable outcome of chemotherapy is tissue damage or organ necrosis, which is usually associated with an increased level of non-functional plasma enzymes, such as AST, ALT, and ALP. These enzymes have diagnostic purposes [41], and they are produced by the liver and heart cells and only increase in the blood when these organs are damaged or suffer tissue necrosis [42].

In our study, the levels of ALT and AST enzymes were significantly improved in mice treated with compound **4**, showing its anti-tumor effects with fewer side effects. It is known that the acute toxicity of compound **4** as a derivative of 2-thioxoimadazolidin-4-one has shown that these substances, according to the classification of Sidorov K.K. [43], are related to substance V1 classes of toxicity, which indicates that they are safe and non-toxic.

## 4. Materials and Methods

### 4.1. Chemistry of Compounds ***1**–**12***

Twelve 2-thioxoimadazolidin-4-one derivatives (**1**–**12**) (Supplementary Table S1) were previously synthesized and characterized by A. I. Khodair according to the modification of the published methods, which are listed in the previous publications [44,45,46,47,48,49,50,51] and the present study Section 2.2. MTT Assay for Cytotoxic Screening.

### 4.2. MTT Assay for Cytotoxic Screening

The in vitro anti-cancer activity was assessed using the MTT assay. We tested the cytotoxic activity of twelve 2-thioxoimadazolidin-4-one derivatives in the HepG2 cell line (human hepatocellular carcinoma cell line), and the most effective compounds were further tested against normal liver cells (THLE-2) to explore their selective action. Staurosporine and 5-FU were used as reference drugs [52]. HepG2 cells (1 × 104 cells/well, 96-well plate) were maintained in DMEM media supplemented with 2mML-glutamine (Lonza, Verviers, Belgium) and 10% FBS (Sigma, St. Louis, MO, USA), 1% Penicillin/Streptomycin (Lonza, Verviers, Belgium) according to the routine culture work [53]. Cultures were incubated for 48 h with serial dilutions (0.01, 0.1, 1, 10, 100 µM) of the tested compounds. Finally, cell viability was determined by measuring the absorbance λ570 nm according to the following formula: % of cell viability = (A sapmple/A control) ×100, then IC_50_ calculation was calculated using GraphPad Prism 7 software [54,55]. We selected compound **4** for further investigation.

### 4.3. Colony Forming Assay

HepG2 cells were allowed to attach to form a complete monolayer after seeding 200 cells per well (6-well plate) for 24 h. Five concentrations of compound **4** (0.01, 0.1, 1, 10, 100 µM) were added to the cultured cells, and cells were incubated for 48 h, followed by washing with PBS. After 7 days of incubation, the colonies were fixed and stained with 1% crystal violet in methanol for 3 h. Finally, the crystal violet stain was used for 20 min to stain and count the cells using the light microscope [56].

### 4.4. Apoptosis Investigation Assays

#### 4.4.1. Flow Cytometric Analysis

Apoptotic cells were quantified using double staining with annexin V and PI on flow cytometry. Compared with untreated cells, HepG2 cancer cells were treated with the corresponding IC_50_ concentration of compound **4** (IC_50_ = 0.017 μM, 48 h); then, as previously mentioned, flow cytometric analysis was performed to investigate the apoptotic cell percentage of action [57,58]. The detailed methodology of flow cytometric analysis (annexin V/PI differential apoptosis/necrosis, DNA content–flow cytometry aided cell cycle) is provided in the Supplementary Materials.

#### 4.4.2. Gene Expression (RT-PCR) Analysis

The target genes of P53, PUMA, Caspases 3, 8, and 9, Bcl-2, PI3K, and AKT were chosen according to the interactive functional association network of the tested protein set using String bioinformatics database for analysis as shown in Supplementary Figure S1. HepG2 cells were treated for 48 h at 37 °C with the IC_50_ value of the compound **4** and followed by real-time *PCR* analysis for control and treated cells of the following genes, P53, PUMA, BCL2, CASP3, CASP8, CASP9, PI3k, and AKT, using the specific primers mentioned in Supplementary Table S3 compared to the control cells. Relative gene expression was calculated utilizing the ΔΔCT equation with normalization to the housekeeping beta-actin gene. Results were shown as a fold change in the mentioned gene expression in the treated versus control cells [55,57,58].

#### 4.4.3. Western Blot

Protein expression analysis for phosphorylated/total phosphatidylinositol 3-kinase (PI3K), protein kinase B (Akt), Caspase 3 and 9 proteins, and Bcl-2 was performed using the Western blotting technique to confirm the mechanism of apoptosis induction by compound **4** in HepG2 cells compared to control. Detailed methodology is supported in Supplementary Materials.

### 4.5. In Vivo Assay

#### 4.5.1. Animals

We used adult Swiss albino female mice with average body weight 18–23 g obtained from Theodor Bilharzia Research Institute in Giza, Egypt. Mice were kept under constant conditions of a 12 h light–dark cycle at temperatures below controlled moisture conditions (22 ± 2 °C) with standard laboratory mouse nutrition and water entries. Approval for the experimental protocol was provided from the ethical committee of Suez Canal University for this experimental protocol (approval number REC-06-2020, Faculty of Science, Suez Canal University). Mice were adapted for approximately 10 days before use in the experiment.

#### 4.5.2. Median Lethal Dose (LD50)

Adopting the procedure described by Finney [59], different known concentrations of the imidamide 4 were intraperitoneally injected into a group of eight mice (weight on average 21.1 g). The LD50 was estimated by recording the 24 h mortality and was found to be 6 mg/kg (I.p.). This assay was carried out evaluate its dose-safety limit and select the dose to be used in the treatment.

#### 4.5.3. Tumor Cell Line and Transplantation

Solid Ehrlich carcinoma (SEC) was obtained from the National Cancer Institute (University of Cairo, Egypt). The tumor cell line proliferated in mice after serial implantation of the peritoneal cavity (I.P.) in 0.2 mL of physiological saline containing 1 × 106 viable cells for 24 h. After 7 days, SEC cells were harvested and diluted with saline to give a 5 × 10^6^ viable SEC cells/mL concentration. An amount of 0.2 mL of saline containing 1 × 10^6^ SEC cells were implanted intraperitoneally in each normal mouse. SEC cells (1 × 10^6^ tumor cells/mouse) were constituted subcutaneously within the right thigh of the hind limb.

#### 4.5.4. Experimental Design

We randomly divided 50 mice into groups 5 groups (10 mice in each group): normal control (I) (mice injected intraperitoneally with the same volume of solvent for 14 consecutive days); group II, a group of normal mice treated with compound **4** (mice injected intraperitoneally with newly produced compound **4** at a dose of 6 mg/kg per day for 14 days); group III, solid tumor group (mice were implanted with solid tumor); group IV, group were treated with 5-FU (after 10 days of tumor implantation, mice were treated with 6 mg/kg only once); and group V, solid tumor group were treated with compound **4** (after 10 days of tumor implantation, mice were treated with 6 mg/kg only once). after one day of the previous treatment, 10 mice from each group were sacrificed and tumor weight, tumor volume (length and width of solid femoral tumor), blood and liver samples were obtained from each group. A portion of each blood sample taken was used for complete blood count (CBC) and the rest was centrifuged to obtain the serum. Serum was used for biochemical parameter estimation. The livers were rapidly dissected, washed with isotonic saline, and dried. The experiment design is summarized in Supplementary Figure S3.

#### 4.5.5. Tumor Volume and Tumor Inhibition Ratio (TIR%)

We measured the length and width of the tumor using a digital Vernier clipper (Tricle Brand, Shanghai, China), then we calculate the tumor volume using the formula: V = (L × W × W)/2, where V is tumor volume, W is tumor width, and L is tumor length, and calculated the tumor inhibition ratio% using the formula: TIR %= (C − T)/C × 100, where T represents mean tumor volume of treated group and C represents mean tumor volume of the control group.

#### 4.5.6. Hematological Assays

Complete blood counts (CBCs) were counted using an AbbottCELLDYN^®^1800 Automated Hematology Analyzer (Abbott Park, IL, USA) equipped with an off-the-shelf kit (Abbott Laboratories, Abbott Park, IL, USA). For microscopic histopathological examination, tumor and kidney slices were maintained in 10% formalin, dehydrated with graduated alcohol, embedded in paraffin sections, H&E stained, and hematoxylin stained.

#### 4.5.7. Serum Biochemical Parameters

Evaluation of the activities of aspartate aminotransferase (AST) and alanine aminotransferase (ALT) using a commercial kit (ELITech Clinical Systems, France). The serum albumin level was measured using a kit obtained from STANBIO Company (USA). A commercial kit from the BioDiagnostic Company (Cairo, Egypt) was used to evaluate creatinine and urea levels.

#### 4.5.8. Oxidant and Antioxidant Assessment

In liver tissue homogenate, a standard commercial kit (BioDiagnostic, Cairo, Egypt) was used to evaluate oxidation and antioxidant parameters according to its methodology, as previously published in Nafie et al., 2020 [57]. Lipid peroxidation (MDA) and nitric oxide (NO) are oxidants, while reduced glutathione (GSH), catalase (CAT), and superoxide dismutase (SOD) are antioxidants.

#### 4.5.9. Histopathological Study

Specimens from mice liver cells were fixed in 10% formalin saline. Then, the samples were dehydrated with ascending series of ethanol and embedded in paraffin. A 5 mm thick section was stained with hematoxylin and eosin and examined under a light microscope.

### 4.6. In Silico Studies

#### 4.6.1. Molecular Docking

The probable mechanism of interaction was demonstrated through molecular docking towards the PI3k/AKT proteins. The molecular docking work was carried out using routine work following Nafie et al., 2019 [60] from ligands and receptors (PI3K (PDB = 1E7V) and AKT (PDB = 3QKK)) preparation, optimization, binding site identification, molecular docking calculation, and finally, analysis of ligand–receptor interactions. Derivatives tested were 3D via the MOE Program Builder interface, and a partial charge was automatically calculated with energy minimization with the MMFF94X force. The verification process was achieved by re-docking the co-crystallized ligand into the active site using the default settings. Different conformers for each compound were introduced by systematic conformational of the MOE and saved in an MDB database file to be docked into the receptor’s active site. Finally, Chimera software was used to imagine and analyze the interactions of the docked compounds and protein.

#### 4.6.2. ADME Pharmacokinetics

A group of web servers, including “MolSoft,” “Molinspiration,” and “SwissADME,” were used to calculate in silico ADME pharmacokinetics parameters of the most active compounds as previously described [60,61].

### 4.7. Statistical Analysis

Data are expressed as a mean value for three different replicates (unless otherwise noted), with a standard error of the mean (SEM). All statistical analyses were performed using SPSS Version 22. Statistical differences between the two groups were investigated using the Student’s *t*-test, and one-way ANOVA was used when testing two or more groups. The significance level was set at *p* < 0.05.

## 5. Conclusions

Taken together, the PI3K/AKT pathway plays an essential role in HepG2, and its inhibition represents a practical approach for liver cancer treatment. This work sheds light on the chemotherapeutic activity of 2-thioxoimadazolidin-4 derivatives (compound **4**) as promising apoptotic anti-liver cancer agents through PI3K/AKT inhibition signaling pathways. Compound **4** exhibited promising cytotoxic activity against the HepG2 cells with an IC_50_ value of 0.017 µM; it induced apoptotic cell death in HepG2 cells in a dose–response manner, arresting the cell cycle G2/M phase. The PI3K/AKT inhibitory signaling pathway was validated as the effective molecular target for the tested compound through gene expression levels and silico levels. Additionally, in the in vivo results, compound **4** inhibited the tumor proliferation by 48.4% compared to 29.28% for 5-FU, activated antioxidants (CAT, SOD and GSH) levels, and ameliorated hematological and biochemical parameters, as well as histopathological examinations. Therefore, compound **4** is recommended to be further developed as an anti-liver cancer agent.

## Figures and Tables

**Table 1:** Summarized IC_50_ values for the activity of the twelve 2-thioxoimadazolidin-4-one compounds **1**–**12** against (HepG2) and (THLE-2) using the MTT assay.

Compounds	IC_50_ *^,#^ ± SE (µM)	
	HepG2 Liver Cancer Cells	THLE-2 Normal Liver Cells
**1**	27.82 ± 0.94	-
**2**	0.18 ± 0.03	16.87 ± 1.11
**3**	8.93 ± 0.75	-
**4**	0.017 ± 0.004	56.55 ± 1.5
**5**	1.94 ± 0.09	-
**6**	0.61 ± 0.08	35.14 ± 1.33
**7**	0.41 ± 0.009	15.36 ± 0.74
**8**	0.79 ± 0.04	14.10 ± 0.58
**9**	**NA**	-
**10**	64.78 ± 1.63	-
**11**	1.14 ± 0.05	-
**12**	1.13 ± 0.066	-
**^#^ Staurosporine**	5.07 ± 0.22	17.6 ± 0.94
**5-FU**	5.18 ± 0.53	42.98 ± 1.29

* Values are expressed as mean ± SE of three independent triplets (*n* = 3). **NA**: non-active. ^#^ IC_50_ was calculated using GraphPad Prism **7** software with nonlinear regression Dose-Inhibition curve fit. **^#^** Staurosporine and 5-FU were chosen as the reference drugs.

**Table 2:** Effects of compound **4** and 5-FU treatments on the hematological parameters of SEC-bearing mice.

Parameters	Treatments					*p*-Value
	Normal Control	Normal + Compound 4 (6 mg/Kg BW)	SEC Control	SEC + Compound 4 (6 mg/Kg BW)	SEC + 5-FU	
Hemoglobin (g/dL)	13.3 ± 0.7	12.6 ± 0.5	11.8 ± 0.8	13.3 ± 0.7	10.6 ± 1.1	0.18
RBC’s count (×10^6^/µL)	8.8 ± 0.3	8.7 ± 0.4	7.52 ± 0.5	9.3 ± 0.4	7.5 ± 0.8	0.12
WBC’s count (×10^3^/µL)	9.4 ± 1.2	9.42 ± 1.3	16.5 ± 3.4	9.5 ± 1.9	13.1 ± 4.1	0.009 *

Results are expressed as mean ± SE (*n* = 9). * Significant difference between groups *(p* ≤ 0.05) by ANOVA followed by Bonferroni post hoc test using SPSS Version 22.

**Table 3:** Effects of compound **4** and 5-FU treatments on the biochemical parameters of SEC-bearing mice.

Parameters	Treatments					*p*-Value
	Normal Control	Normal + Compound 4 (6 mg/Kg BW)	SEC Control	SEC + Compound 4 (6 mg/Kg BW)	SEC + 5-FU	
Urea (mg/dL)	27.8 ± 2.6	28.13 ± 2.7	25.2 ± 2.3	27 ± 1.5	26.1 ± 2.7	0.38
Creatinine (mg/dL)	1.6 ± 0.2	1.1 ± 0.2	0.7 ± 01	1.0 ± 0.1	0.8 ± 0.1	0.16
ALT (U/L)	35.3 ± 3.8	43.25 ± 7.4	50 ± 2.7	37.4 ± 10.4	39.1 ± 3.6	0.22
AST (U/L)	37.4 ± 2.8	44.9 ± 10.8	48.9 ± 5.5	37.8 ± 8.4	38 ± 4.6	0.42
Total Protein (g/dL)	6.9 ± 0.4	6.9 ± 1.5	6.6 ± 0.4	7.4 ± 0.2	7.2 ± 0.4	0.39
Albumin (g/dL)	4.4 ± 0.4	4.4 ± 0.8	4.01 ± 0.4	3.43 ± 0.1	4.1 ± 0.3	0.35

Results are expressed as mean ± SE (*n* = 9), and the statistical test was computed using ANOVA followed by Bonferroni post hoc test in SPSS Version 22.

**Table 4:** Assessment of hepatic oxidant/antioxidant activities in the studied groups of SEC-bearing mice.

Treatments	Oxidative Stress		Antioxidants		
	MDA (nmol/g Tissue)	NO (U/g Tissue)	GSH (mg/g Tissue)	CAT (U/g Tissue)	SOD (U/g Tissue)
Normal control	61.38 ± 2.07	36.58 ± 1. 7	47.23 ± 2.08	53.35 ± 2.01	46.39 ± 2.08
SEC control	98.39 * ± 2.04	79.69 * ± 2.58	18.23 * ± 1.09	21.69 * ± 0.87	27.36 * ± 1.27
SEC+ Compound **4** (6 mg/kg BW)	73.36 ^#^ ± 2.15	42.38 ^#^ ± 1.08	39.27 ^#^ ± 1.38	46.9 ^#^ ± 1.87	48.69 ^#^ ± 0.95

Results are expressed as mean ± SE (*n* = 6). * Significant difference between SEC control and normal control using an unpaired *t*-test (*p* ≤ 0.05). ^#^ Significant difference between SEC control and compound **4** SEC-treated groups using an unpaired *t*-test (*p* ≤ 0.05) using GraphPad prism.

**Table 5:** Analysis of ligand–receptor interactions with binding energies of docked compounds **4** towards PI3K and AKT proteins.

Compound	PI3k (1E8Z) *			AKT (3QKK) *		
	Binding Energy	HB Interaction	Other Interactions	Binding Energy	HB Interaction	Other Interactions
**4**	−18.58 Kcal/mol	1 Hydrogen bond with **Val 882**	Arene–arene interactions with Tyr 867 and arene-cation with Lys 890	−10.97 Kcal/mol	1 Hydrogen bond with **Asp 292**	-

* Docking calculations with MOE software were validated by RMSD calculation ≤ 1.5.

**Table 6:** In silico ADME pharmacokinetics properties.

#	Molinspiration 2018.10						MolSoft			Swiss ADME
	MWt (D)	MV (A^3^)	PSA (A^2^)	Log P	Nrotb	Nviolations	HBA	HBD	Solubility (mg/L)	Drug Likeness (Lipinski Pfizer Filter)
**1**	310.38	269.60	47.03	2.48	3	0	3	1	52.40	“Yes, drug-like” MW ≤ 500, Log P ≤ 4.15, HBA ≤ 10 and HDD ≤ 5
**2**	319.39	273.04	53.59	2.88	2	0	2	2	38.07	
**3**	261.35	235.12	41.03	1.25	2	0	2	1	303.25	
**4**	302.38	242.79	58.02	1.84	2	0	4	2	450.13	
**5**	375.45	325.26	57.88	0.98	4	0	6	0	2413.05	
**6**	258.35	233.74	37.08	2.24	3	0	2	1	68.95	
**7**	286.38	234.77	37.80	2.32	2	0	3	1	138.71	
**10**	257.36	235.54	44.81	0.69	3	0	4	1	3509.58	
**11**	352.55	345.78	38.81	2.76	5	0	4	1	165.26	
**12**	369.53	351.00	54.04	2.50	6	0	5	1	355.76	

“MWt: Molecular Weight, MV: Molecular Volume, PAS: Polar Surface Area, Log P: Octanol-water partition coefficient, nrotb: number of rotatable bond, nviolations: number of violations, HBA: Hydrogen Bond Acceptor, HBD: Hydrogen Bond Donor”.

## Data Availability

The datasets used in the current study are available from the corresponding authors on reasonable request.

## Supplementary Materials

The following are available online, Table S1: Some derivatives of 2-thioxoimidazolin-4-one (**1**–**12**); Table S2: Quantification data of the H&E analysis of liver and kidney; Table S3: Forward and reverse primers used in gene expression analysis. Figure S1: Interactive functional association network of the proteins set (Bcl2, Casp9, PIK3, and AKT); Figure S2: BOILED Egg model for compound **4** using the Swiss-ADME. Figure S3: In vivo assay summary.
