# Supplementary material for: Evaluation of 2-Thioxoimadazolidin-4-one Derivatives as Potent Anti-Cancer Agents through Apoptosis Induction and Antioxidant Activation: In Vitro and In Vivo Approaches
Source: Molecules. 2021 Dec 23;27(1):83. doi: 10.3390/molecules27010083 (PMC8746798; doi:10.3390/molecules27010083)
Supplement: Supplementary file 1 [file molecules-27-00083-s001.zip › molecules-1475997-supplementary.pdf]

molecules

Supplementary materials for detailed methodology

Evaluation of 2-thioxoimadazolidin-4-one Derivatives as Potent Anti-cancer Agents Through Apoptosis Induction and Antioxidant Activation: *In vitro* and *In vivo* Approaches

Mohamed S. Nafie [1,*], Ahmed I. Khodair [2,*], Hebat Allah Y. Hassan [3], Noha M. Abd El-Fadeal [4], Hanin A. Bogari [5], Sameh S. Elhady [6] and Safwat A. Ahmed [3,7,*]

[1] Department of Chemistry, Faculty of Science, Suez Canal University, Ismailia 41522, Egypt
[2] Department of Chemistry, Faculty of Science, Kafrelsheikh University, 33516, Kafrelsheikh, Egypt
[3] Institute of Biotechnology for Graduate Studies & Research, Suez Canal University, Ismailia 41522, Egypt; omrelansary2010@gmail.com
[4] Department of Medical Biochemistry and Molecular Biology, Faculty of Medicine, Suez Canal University, Ismailia 41522, Egypt; noha_abdelfadeal@med.suez.edu.eg
[5] Department of Pharmacy Practice, Faculty of Pharmacy, King Abdulaziz University, Jeddah 21589, Saudi Arabia; hbogari@kau.edu.sa
[6] Department of Natural Products, Faculty of Pharmacy, King Abdulaziz University, Jeddah 21589, Saudi Arabia; ssahmed@kau.edu.sa
[7] Department of Pharmacognosy, Faculty of Pharmacy, Suez Canal University, Ismailia 41522, Egypt
* Correspondence: mohamed_nafie@science.suez.edu.eg (M.S.N.); khodair2020@yahoo.com (A.I.K.); safwat_ahmed@pharm.suez.edu.eg (S.A.A.)

Flow cytometric analysis

FITC/Annexin-V-FITC/PI Differential Apoptosis/Necrosis Assessment

Apoptosis and necrosis cell populations are determined using Annexin V-FITC apoptosis detection kit (Abcam Inc., Cambridge Science Park, Cambridge, UK) coupled with 2 fluorescent channels flowcytometry. After treatment with test compounds for 48 h, cells (10^5 cells) are collected by trypsinization and washed twice with ice-cold PBS (pH 7.4). Then, cells are incubated in dark with 0.5 ml of Annexin V-FITC/PI solution for 30 min in dark at room temperature according to manufacturer protocol. After staining, cells are injected via ACEA Novocyte™ flowcytometer (ACEA Biosciences Inc., San Diego, CA, USA) and analyzed for FITC and PI fluorescent signals using FL1 and FL2 signal detector, respectively (λex/em 488/530 nm for FITC and λex/em 535/617 nm for PI). For each sample, 12,000 events are acquired and positive FITC and/or PI cells are quantified by quadrant analysis and calculated using ACEA NovoExpress™ software (ACEA Biosciences Inc., San Diego, CA, USA).

DNA Content-Flow Cytometry Aided Cell Cycle Analysis

After treatment with test compounds for 48 h, cells (10^5 cells) are collected by trypsinization and washed twice with ice-cold PBS (pH 7.4). Cells are re-suspended in two milliliters of 60% ice-cold ethanol and incubated at 4ºC for 1 h for fixation. Fixed cells are washed twice again with PBS (pH 7.4) and re-suspended in 1 mL of PBS containing 50 µg/mL RNAase A and 10 µg/mL propidium iodide (PI). After 20 min of incubation in dark at 37 C, cells are analyzed for DNA contents using flow cytometry analysis using FL2 (λex/em 535/617 nm) signal detector (ACEA Novocyte™ flowcytometer, ACEA Biosciences Inc., San Diego, CA, USA). For each sample, 12,000 events are acquired. Cell cycle distribution is calculated using ACEA NovoExpress™ software (ACEA Biosciences Inc., San Diego, CA, USA).

Gene expression using RT-PCR

HepG2 cells were treated for 48 h at 37 °C with the IC_{50} value of the compound **4** and were collected for real-time PCR analysis with the perfect primer pairs (Supplementary, Table 1) of the following eight genes (P53, PUMA, BCL2, CASP3, CASP8, CASP9, PI3k and AKT) compared to the control cells, and for analysis first total RNA was extracted using RNeasy Mini Kit (Qiagen) following the manufacturer protocol of the kits than RNA was assessed for both the quality and quantity of RNA using NanoDrop ND-1000 spectrophotometer at the absorbance ratio 260/280 nm [NanoDrop Tech., Inc. DE, USA]. Also, Whole RNA quality was examined by 1% agarose gel electrophoresis and afterward cDNA was obtained from one µg RNA using I-Script cDNA synthesis kit (BioRad). qRT-PCR were executed utilizing 10 ng of cDNA and Applied Biosystems Sybr Green master mix [USA]. Primers utilized during real time PCR was listed in table (1) and the cycling conditions were 5 min [95 °C], followed by 45 cycles for 15 s [95 °C], for 60 s [58, 60, 62 °C] and for 1 min [72 °C] using applied Biosystems Step-One qRT-PCR (USA). Relative genes expression was finally calculated utilizing the ΔΔCT equation with normalization to that of the housekeeping beta-actin gene. Results were shown as fold change in gene expression in the treated cells versus that of the control**.**

Table S1. Some derivatives of 2-thioxoimidazolin-4-one (1-12).

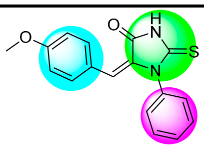 (*E*)-5-(4-Methoxybenzylidene)-1-phenyl-2-thioxoimidazolidin-4-one (**1**)	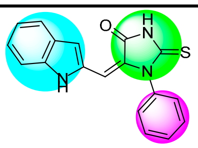 (*E*)-5-((1*H*-Indol-2-yl) methylene)-1-phenyl-2-thioxoimidazolidin-4-one (**2**)
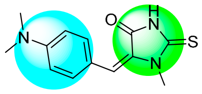 (*E*)-5-(4-(Dimethylamino) benzylidene)-1-methyl-2-thioxoimidazolidin-4-one (**3**)	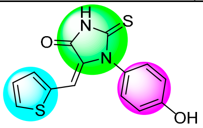 (*E*)-1-(4-Hydroxyphenyl)-5-(thiophen-2-ylmethylene)-2-thioxoimidazolidin-4-one (**4**)
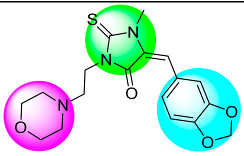 (*E*)-5-(3,4-Methylenedioxybenzylidene)-3-[2-(4-morpholino) ethyl]-1-methyl-2-thioxo-imidazolidin-4-one (**5**)	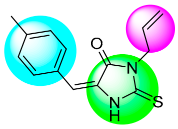 (*E*)-3-Allyl-5-(4-methylbenzylidene)-2-thioxoimidazolidin-4-one (**6**)
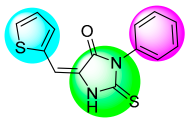 (*E*)-3-Phenyl-5-(thiophen-2-ylmethylene)-2-thioxoimidazolidin-4-one (**7**)	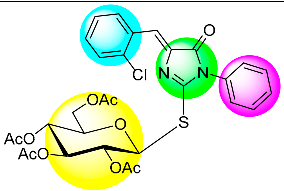 5-(4-Chlorobenzylldene-3-pheny1-2-(2',3',4',6'-tetra-*O*-acetyl- -D-glucopyranosyl)-2-thioxoimidazolidin-4-one (**8**)
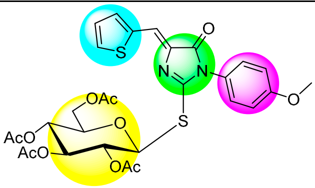 3-(4-Methoxyphenyl)-5-(2-thiophen-2-ylmethylene)-2-(2',3',4',6'-tetra-*O*-acetyl- -D-glucopyranosyl)-2-thioxoimidazolidin-4-one (**9**)	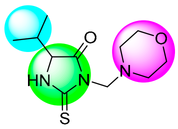 5-Isopropyl-3-(morpholinomethyl)-2-thioxoimidazolidin-4-one (**10**)

5-Isopropyl-3,5-bis(piperidin-1-ylmethyl)-2-thioxoimidazolidin-4-one (**11**)	5-Isobutyl-3,5-bis(morpholinomethyl)-2-thioxoimidazolidin-4-one (**12**)

Table S2. Forward and reverse primers used in gene expression analysis.

Gene	Forward	Reverse
**P53**	5'- CCCCTCCTGGCCCCTGTCATCTTC-3'	5'- GCAGCGCCTCACAACCTCCGTCAT -3'
**PUMA**	5′-GAGGAGGAACAGTGGGC-3'	5′-CTAATTGGGCTCCATCTCGG-3′
**CASP-3**	5'- TGGCCCTGAAATACGAAGTC-3'	5'- GGCAGTAGTCGACTCTGAAG -3'
**CAS-8**	5'- AATGTTGGAGGAAAGCAAT -3'	5'- CATAGTCGTTGATTATCTTCAGC -3'
**CASP-9**	5'- CGAACTAACAGGCAAGCAGC -3'	5'- ACCTCACCAAATCCTCCAGAAC -3'
**BCL2**	5’- CCTGTGGATGACTGAGTACC -3’	5’- GAGACAGCCAGGAGAAATCA -3’
**PI3K**	5'-CTCTCCTGTGCTGGCTACTGT-3`	5'-GCTCTCGGTTGATTCCAAACT-3'
**AKT**	5'-GGACAAGGACGGGCACATTA-3'	5'-CGACCGCACATCATCTCGTA-3'
**β-actin**	5’- GTGACATCCACACCCAGAGG -3`	5’- ACAGGATGTCAAAACTGCCC -3’

Interactive functional association network of the proteins set (Bcl2, Casp9, PIK3 and AKT1).

As seen in **Fig. S1**, the interactive functional association network of the tested protein set using String bioinformatics database for analysis.

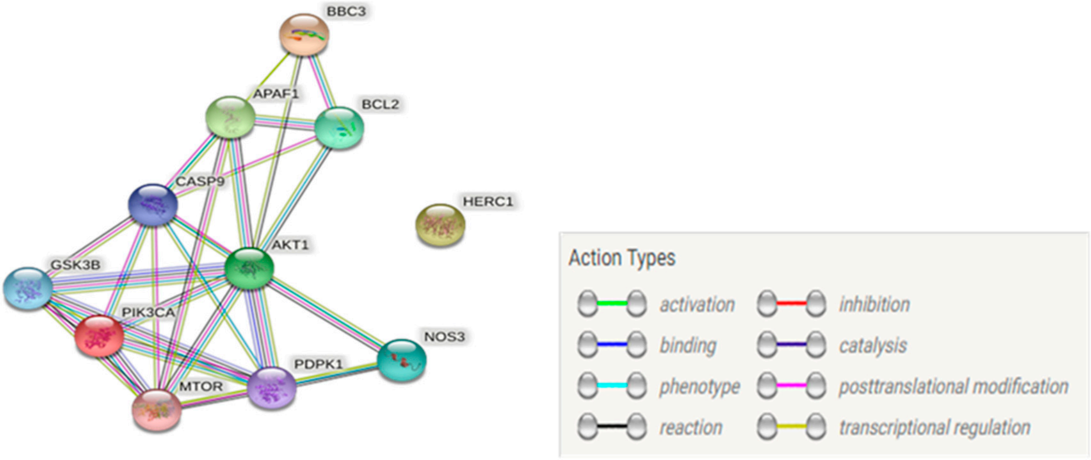

Figure S1. Interactive functional association network of the proteins set (Bcl2, Casp9, PIK3 and AKT1). The network nodes are proteins. Predicted functional links between the proteins are indicated by edges in color-coded lines. To illustrate, the network String bioinformatics database for this analysis was used (https://stringdb.org).

ADME pharmacokinetics

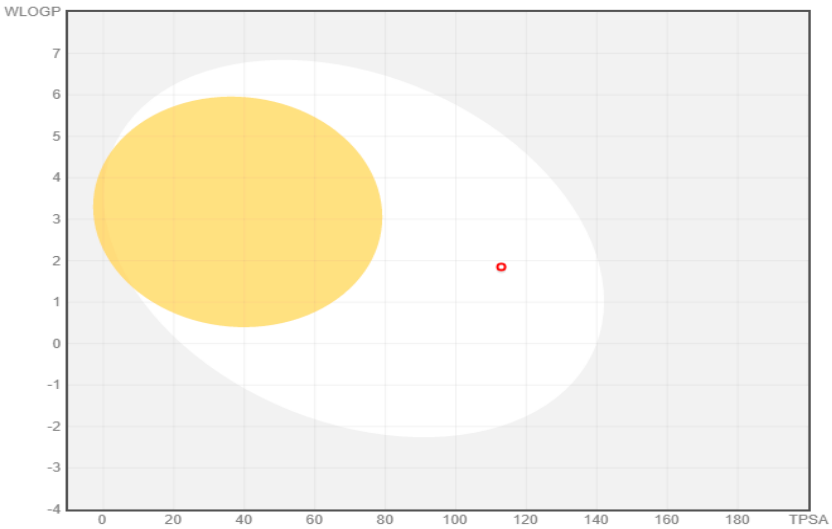

Figure S2. BOILED-Egg model for compounds **4** using the SwissADME. "Points located in the BOILED-Egg`s yolk are molecules predicted to passively permeate through the blood–brain barrier, while points located in the BOILED-Egg`s white are molecules predicted to be passively absorbed by the gastrointestinal tract.".

In Vivo Assay

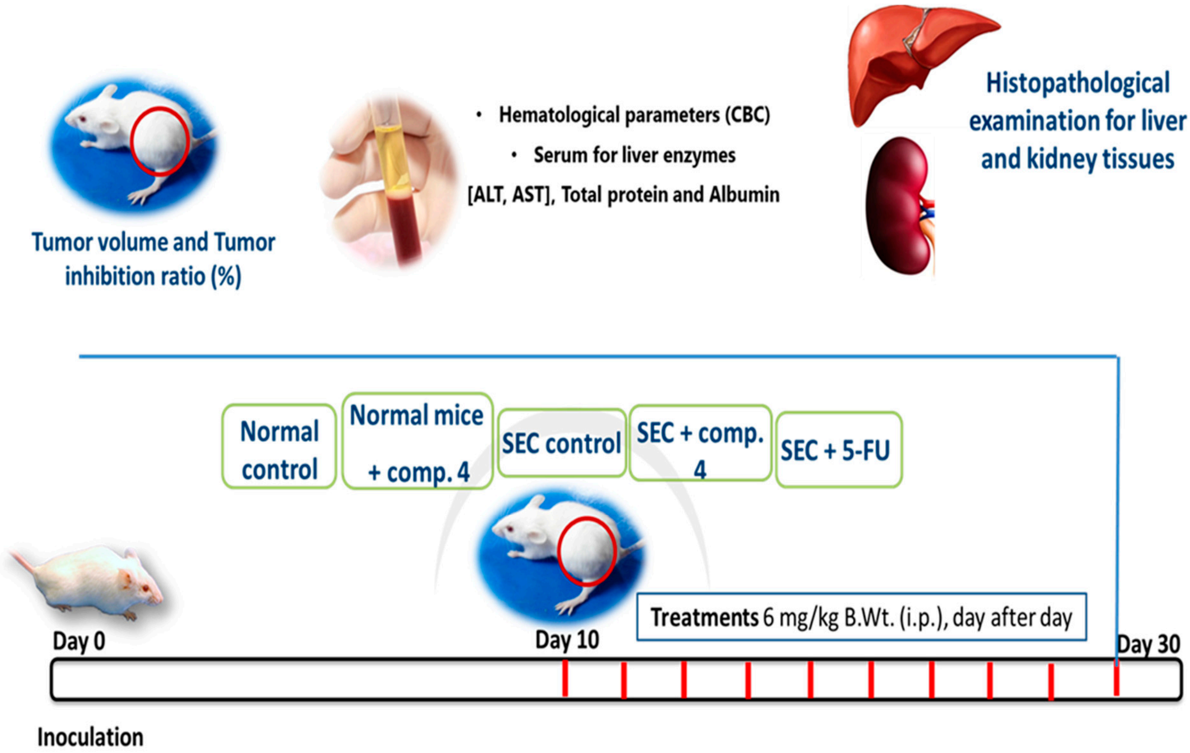

Figure S3. In Vivo Assay Summary.
